# Perceptions of a Pragmatic Family-Centered Approach to Childhood Obesity Treatment

**DOI:** 10.31486/toj.19.0126

**Published:** 2021

**Authors:** Betty M. Kennedy, Genevieve Davison, Lauren A. Fowler, Erika Rodriguez-Guzman, Myra L. Collins, Alison Baker, Stephen Cook, Jeanne Lindros, Denise E. Wilfley, Ava J. Zebrick, Amanda E. Staiano

**Affiliations:** ^1^ Pennington Biomedical Research Center, Baton Rouge, LA; ^2^ Department of Psychiatry, Washington University School of Medicine, St. Louis, MO; ^3^ American Academy of Pediatrics, Itasca, IL; ^4^ Department of Research, Golisano Children's Hospital, University of Rochester Medical Center, Rochester, NY

**Keywords:** *Pediatric obesity*, *primary health care*, *weight loss*, *weight reduction programs*

## Abstract

**Background:** Few studies have examined both parent and child preferences regarding family-based weight management programs (WMPs) delivered in primary care settings, especially among racial minority populations. The purpose of this study was to determine the perceptions that parents and their children/adolescents have about the components that should be included in a family-based WMP and to identify perceived preferences, benefits, and/or barriers to participation.

**Methods:** A sample of 60 participants (30 parents and 30 children/adolescents) participated in 1 of 5 separate structured focus groups, using probing questions and the nominal group technique (NGT). Parents reported demographics for themselves and their children/adolescents. Themes from probing questions were identified using thematic analysis.

**Results:** Parents were primarily African American (93%) and diverse in income. NGT sessions revealed that parents across all groups perceived that education on healthy eating, parental involvement, and effective program leaders are most important and have the greatest impact, while parental involvement was perceived as the easiest method to implement in a family-based WMP for childhood obesity. Children/adolescents perceived that education on healthy eating and exercise would have the greatest impact, while healthy eating and meal plans were perceived as the easiest methods to implement with a family. Parents and children/adolescents also identified improved psychological well-being (eg, decreased bullying, increased self-esteem, and motivation) as a desired program outcome.

**Conclusion:** Parents and their children/adolescents highlighted the importance of physical and psychological health as targets in treatment. Feedback from patients can inform the design and implementation of family-based WMPs delivered in primary care settings.

## INTRODUCTION

Almost 1 in 5 youth (18.5%) struggles with obesity,^1,2^ and 9.5% of adolescents have severe obesity (body mass index [BMI] ≥120% of the 95th percentile, or ≥35 kg/m^2^).^3^ Treatment of childhood obesity is complicated by complex and multifactorial etiology, with a multitude of contributing factors including genetics, developmental effects, fetal programming and epigenetics, environment, behavioral and psychosocial issues, physical activity, medications, eating patterns, illness, and cultural and family norms.^4^ Additionally, many children and adolescents with obesity have lower health-related quality of life compared to children and adolescents with normal weight and suffer from harmful psychosocial stigma.^5-7^

Primary care providers (PCPs) are tasked with periodic screening of children's health and growth, and as a result, PCPs play a vital role in recognizing and addressing childhood obesity, a condition associated with substantial health and economic burdens.^8-11^ As of 2011, however, active participation from PCPs in assessing or managing children with overweight or obesity in primary care clinics was low,^11^ even though pediatricians can easily identify children with obesity.^12^ Therefore, equipping PCPs with effective prevention and treatment tools to address childhood obesity that can be easily implemented is essential. Qualitative research published in 2016 shows that parents of children with overweight or obesity would like PCPs to take an active role in identifying child overweight or obesity and helping parents manage their children's weight.^13,14^

A common modality of multidisciplinary treatment for children with obesity is family-based weight management programs (WMPs), where parents and children attend sessions with a trained counselor or coach together as a family.^15^ Family-based behavioral treatments (FBTs) that simultaneously target children and their parents are effective ways to promote weight loss and management through healthy eating and exercise.^16-18^ In FBT, parents are taught positive parenting techniques, including contingency management, environmental control, and ways to promote their child's self-regulatory skills through behavioral economics and social learning theory.^19^

Integrating FBTs into primary care settings either by placing trained coaches in primary clinics or training providers in FBT techniques could improve treatment reach and accessibility and provide PCPs with an effective treatment option for pediatric patients with overweight and obesity. Few models for successfully adapting FBTs for non-specialty settings are available,^20^ although some studies show promise. A randomized controlled trial of a family-based weight control program for preschool-aged children implemented in primary care was found to be effective at helping children lose weight.^21^ A pilot study evaluating the acceptability of group-based FBT for older children in primary care found that such programs may be effective and acceptable but noted challenges with both recruitment and retention.^22^ The successful implementation of FBTs for pediatric obesity in primary care settings will require a better understanding of families’ attitudes regarding family-based WMPs.

The purpose of this study was to determine the perceptions parents and their children/adolescents have about the components that should be included in a family-based WMP in a primary care setting and to identify perceived preferences, benefits, and/or barriers to participation. Previous research in this area has largely focused on parent or child/adolescent preferences regarding the role of PCPs in child/adolescent weight management or WMPs,^13,14,23^ but few studies have examined both parent and child/adolescent preferences regarding family-based WMPs delivered in a primary care setting.^24-26^ Information gathered from this study is important to adapt family-based weight loss interventions in PCP settings.

## METHODS

### Participants

Parents and their children/adolescents aged 6 to 17 years old who were able to speak and understand English were eligible to participate in 1 of 5 separate structured focus groups. We did not impose an a priori inclusion criterion based on BMI; rather, we sought participants with an interest in discussing weight loss and weight management strategies. Individuals with a cognitive impairment that would interfere with participation in a group discussion were not eligible.

Participants were recruited principally by word of mouth and flyers printed in church bulletins or distributed at schools. Word-of-mouth recruitment relied on a network of friends, colleagues, neighbors, and relatives of current and previous study participants to spread the word about the study to help us reach our goal. When participants arrived at the focus group location and prior to the start of each discussion, the researcher read aloud a group informed consent that explained all aspects of the focus group that are normally detailed in a written document. The researcher emphasized that participation was voluntary, and those who did not wish to participate were asked to leave the room prior to the start of the focus group discussion. By remaining in the room after the consent document was read, participants signaled their agreement to participate in the study. Light refreshments were provided, and each family received a $50 stipend. The Pennington Biomedical Research Center Institutional Review Board reviewed and approved the study protocol, procedures, and consent form.

### Design and Procedures

The nominal group technique (NGT), a qualitative method of data collection, was used to engage parents and their children/adolescents to obtain their perspective on the components that should be included in family-based WMPs. The NGT is a brainstorming tool for quality improvement and highly structured small group discussions and is used to elicit and prioritize a list of answers to a specific question.^27-31^ Similar to traditional focus groups, 4 to 12 participants per group may be considered appropriate for NGT sessions.^32^

The multistep NGT design is useful for systematically stimulating meaningful interpersonal statements among participants by gathering equally weighted responses to a specific question that tends to offer valid representation of group views.^33-36^ With the NGT method, audio recording and transcription are not necessary because verbatim responses are written on a flipchart, thereby providing a concise summary of the session that is readily available for dissemination. Prior to conducting the NGT sessions, the investigative team articulated the specific question and then pilot tested it with staff members, including those with children having a desire to reduce their weight, to ensure that it would elicit meaningful responses.

Parents and at least one child/adolescent residing in or within an hour of Baton Rouge, Louisiana, participated in 1 of 5 separate NGT sessions. Each focus group consisted of 5 to 7 participants and was conducted on Saturday morning, Saturday at noon, or Thursday evening, beginning at the end of September and ending on the first day of November 2018. Three sessions were conducted at the Pennington Biomedical Research Center, 1 at a charter school, and 1 at an agricultural center in Opelousas, Louisiana. Each group session lasted for approximately 90 minutes.

After welcoming the participants and providing brief introductions, the facilitators discussed the purpose of the session and ground rules for participation. To initiate each session, facilitators discussed probing questions with parents and children/adolescents, including “What are the most important results you’d expect from a weight management program?”; “What would make you want to stick with a program, even if you and your child needed to attend the program every week for several months?”; “What factors might prevent your child and you from participation in a weight management program?”; and “If we are successful in developing curriculum and materials for a family-based weight management program, what would that look like?”

The facilitators (B.M.K., E.R.G.), accompanied by a co-facilitator (M.L.C.), posed the specific question to parents: “We are working with primary care providers to create a program to help treat obesity in children; what important factors should a program include to treat childhood obesity?” The children/adolescents were asked, “What sorts of things could your doctor or a health coach teach you to help you have a healthy weight?” In response to the specific question, the participants were asked to work silently and independently and to write down as many responses in short phrases as possible that represented their individual views. In a round-robin manner, the participants were then asked to share their answers (one response at a time) while the co-facilitator wrote each response verbatim on a flipchart without discussion. Each recorded response was discussed for the sole purpose of clarification and not for evaluation or debate as to its relative importance. During this step, the participants were asked to combine responses that were perceived to be significantly similar. Finally, during the voting phase, the parents and children/adolescents privately selected what they considered to be the top 3 items from the generated list of responses likely to have the greatest impact and then did a second separate ranking to identify ideas that would be the easiest to implement for weight management.

Each parent and child/adolescent prioritized their choices on their own and without discussing with others, assigned a rank of 3 to the most important strategy and 1 to the least important strategy and likewise for the easiest to implement. The facilitators recorded the votes on the flipchart in front of all participants and then tallied the votes for each response. A small number of idiosyncratic ideas were discarded, which is a standard procedure in the NGT. The main results were the top 3 strategies identified within each group; the secondary results were all other ideas. Through an iterative process, the facilitators categorized responses into common themes until consensus was obtained.

To identify other unique responses to focus group questions and to ensure that nuances in response were recognized given the exploratory nature of the study, the raw data for all probing and specific questions were coded. Specifically, authors G.D. and L.A.F. independently coded the facilitators’ summarized responses from parent and child/adolescent focus groups to identify common themes in responses. Differences in coding of themes among coders were discussed, and consensus of thematic categories for each question was reached.

Demographic information was collected from parents using survey instruments that included age, ethnicity, sex, education, employment, annual household income, and marital and health status. In addition, parents self-reported their own height and weight and the height, weight, and grade level of their child/adolescent.

## RESULTS

Selected demographic characteristics of the 60 focus group participants (30 parents, 30 children/adolescents) are shown in Table 1. The average BMI for parents was 34.7 kg/m^2^ (range, 20.1 to 55.3 kg/m^2^). Fifty percent of children/adolescents had a BMI >95th percentile.

**Table 1.** Characteristics of Participants in the Focus Groups

Variable	Parents, n=30		Children/Adolescents, n=30	
Age, years				
18-35	4 (13)		Not available	
36-55	25 (83)			
56-65	1 (3)			
Race				
Black	28 (93)		Not available	
White	2 (7)			
Sex				
Female	30 (100)		23 (77)	
Male	0		7 (23)	
Education	High school	2 (7)	1st-2nd grade	4 (13)
	1-3 years college	8 (27)	3rd-4th grade	6 (20)
	College degree	10 (33)	5th-6th grade	6 (20)
	Postgraduate degree	10 (33)	7th-8th grade	6 (20)
			9th-10th grade	7 (23)
			11th grade	1 (3)
Employment				
Full-time	26 (87)		Not applicable	
Part-time	2 (7)			
Unemployed	1 (3)			
Retired	1 (3)			
Annual income^a^				
$20,000-$29,999	4 (13)		Not applicable	
$30,000-$39,999	6 (20)			
$40,000-$49,999	6 (20)			
$50,000-$59,999	2 (7)			
$60,000-$69,999	3 (10)			
≥$70,000	9 (30)			
Marital status				
Married	15 (50)		Not applicable	
Divorced/separated	6 (20)			
Never married	9 (30)			
Health status				
Excellent	5 (17)		Not available	
Very good	9 (30)			
Good	14 (47)			
Fair	2 (7)			
Weight status				
Not overweight^b^	3 (10)		15 (50)	
Overweight or obesity^c^	27 (90)		15 (50)	

Note: Data are presented as n (%).

^a^Total household income.

^b^Body mass index <25 kg/m^2^ or <85th percentile.

^c^Body mass index ≥25 kg/m^2^ or ≥85th percentile.

### Nominal Group Technique Sessions–Parents

Thirty parents participated in 1 of 5 NGT sessions and generated 81 responses to the question, *“*What important factors should a program include to treat childhood obesity?” During the clarification discussions, parents within and across all groups indicated that many of the responses were similar or nearly the same, and as a result, responses were amalgamated. The final list for the prioritization exercise consisted of 10 responses that were organized under 4 themes identified during the iterative process: educate on healthy vs unhealthy eating, parental involvement, effective program leaders, and incentives.

Table 2 lists the 4 themes with representative parent responses next to each theme. The relative importance of parents’ suggestions for what should be included in a family-based WMP is reflected by the total number of votes and the sum of ranks. The specific parent responses with the most votes were these: *“*Healthy eating guide to buy healthy foods or training for parents to educate children on how and why certain foods are healthy and not healthy for them”; “Mentor—eat with child; set the example of how to eat healthy”; “Coaching: reassurance no matter your size, no limits”; and “Fun, attractive to kids, and exciting to increase motivation.”

**Table 2.** Parents’ Perceptions of Factors to Include in Family-Based Weight Management Programs (n=30)

Specific question: We are working with primary care providers to create a program to help treat obesity in children; what important factors should a program include to treat childhood obesity?			
Key Themes	Representative Responses	Total Votes	Sum of Ranks^a^
Educate on healthy vs unhealthy eating	“Healthy eating guide to buy healthy foods or training for parents to educate children on how and why certain foods are healthy and not healthy for them.”	19	31
	“Demonstrate healthy ways to eat by having program person prepare and demonstrate that healthy food is good; example of portion size to objects.”	6	18
Parental involvement	“Mentor—eat with child; set the example of how to eat healthy.”	9	27
	“Access to available resources for amount of family activities; low cost/inexpensive or free exercise.”	9	21
	“Program for kid/adults; support each other.”	4	12
Effective program leaders	“Coaching: reassurance no matter your size, no limits.”	9	24
	“Progress evaluation, positive weigh-in for kids.”	7	19
	“Knowledgeable about disease/health consideration related to culture.”	6	15
Incentives	“Fun, attractive to kids, and exciting to increase motivation.”	8	20
	“Gift cards to buy healthy foods.”	5	15

^a^Calculated by summing the ranks of responses (3=most important, 2=second, and 1=least important). Higher score=greater perceived importance.

Parents across all groups perceived the first 3 themes—educate on healthy vs unhealthy eating, parental involvement, and effective program leaders—as most important and likely to have the greatest impact, while parental involvement was perceived as the easiest method to implement in a family-based WMP. Secondary and other suggestions are categorized under each applicable theme displayed in Table 2.

### Nominal Group Technique Sessions–Children/Adolescents

Thirty children/adolescents participated in 1 of 5 NGT sessions and generated 46 responses to the question, “What sorts of things could your doctor or a health coach teach you to help you have a healthy weight?” During the clarification discussions, children/adolescents stated that several responses were repetitive, so responses were combined. The final list for the prioritization exercise consisted of 5 responses that were organized under 2 themes identified during the iterative process: educate on healthy vs unhealthy eating and exercise.

Table 3 lists the 2 themes with representative children/adolescent responses next to each theme. The relative importance of each response for what a doctor or health coach could teach them to have a healthy weight is reflected by the total number of votes and the sum of ranks. The specific children/adolescent responses with the most votes were these: “Recommend a good meal plan for each day per week to have the right nutrients and minerals to maintain a healthy body weight” and “Give you fun activities; play games, basketball, dodgeball, jumping jacks, dancing, and run with you to keep weight off.”

**Table 3.** Children/Adolescents’ Perceptions of How to Have a Healthy Weight (n=30)

Specific question: What sorts of things could your doctor or a health coach teach you to help you have a healthy weight?			
Key Themes	Representative Responses	Total Votes	Sum of Ranks^a^
Educate on healthy vs unhealthy eating	“Recommend a good meal plan for each day per week to have the right nutrients and minerals to maintain a healthy body weight.”	12	30
	“Doctor can tell you what is healthy and not healthy and teach the consequences of not eating healthy.”	6	18
	“We could start training by doing a health plan or diet if our parents are doing the same.”	2	4
Exercise	“Fun activities; play games, basketball, dodgeball, jumping jacks, dancing, and run with you to keep weight off.”	14	28
	“Teach different exercise techniques and ways to work exercise into your schedule.”	6	18

^a^Calculated by summing the ranks of responses (3=most important, 2=second, and 1=least important). Higher score=greater perceived importance.

Children/adolescents across all groups perceived these 2 themes—educate on healthy vs unhealthy eating and exercise—as most important and likely to have the greatest impact, while healthy eating and meal plans were perceived as the easiest methods to implement by a doctor or health coach to teach children/adolescents to have a healthy weight. Secondary and other responses are also noted under the applicable theme in Table 3.

### Themes from Probing Questions

#### Parent Responses

Questions and major themes of parent responses are presented in Table 4. Across all 5 focus groups, parents identified 6 major types of resources that are currently available to them to help their children/adolescents have a healthy weight: the internet; electronic/digital tools and games (eg, “Fitbit” and “Wii Fit”); doctors and specialists, including nutritionists and trainers; school-related resources; private businesses; and community-level resources, including governmental programs (eg, “the library” and “YMCA”).

**Table 4.** Themes From Probing Questions

Parents’ Focus Groups		Children's Focus Groups	
Questions	Themes	Questions	Themes
1. What resources are available to you now to help your child have a healthy weight?	Internet Electronic/digital tools and games Doctors/specialists School Businesses Community (family)/government	1. What are ways that your doctors, teachers, or parents help you have a healthy weight?	Education/feedback Encouragement/advice Engage in activity Monitor weight Influence food intake
2. What are the most important results you’d expect from a weight management program for children?	Increased self-esteem (psychological changes) Weight loss (weight maintenance) Improved health (long-term/lifestyle change) Increased knowledge about health behaviors Behavior change	2. What are the biggest challenges that kids who struggle with having a healthy weight face?	Bullying Physical limitations Behavioral and psychological struggles Lack of knowledge Lack of motivation Lack of self-confidence Tempting food Lack of control over environment
3. What would make you want to stick with a program, even if you and your child needed to attend the program every week for several months?	Results Convenient/flexible Fun Tangible incentives Social support Motivating – Good teacher/coach Child interest	3. We’re working with doctors to create a program to help kids and parents become healthier. If you were in this program, what are the most important results you’d like to see?	Visible results (weight loss) Improved health–mental, physical, fitness, feeling better Reduced bullying Adopt healthy behaviors Increased knowledge
4. We are working with primary care providers to create a program to help treat obesity in children; what important factors should a program include to treat childhood obesity?	Education Skills/behavior change Incentives Fun Culturally appropriate Social support/coaching (family-oriented) Accessible, feasible, sustainable, affordable Use of technology/apps websites	4. What would make you want to stick with a program, even if you needed to go to the program every week after school for several months?	Physical or visible results Better health Learning new skills Better knowledge Intrinsically rewarding–encouragement, games, motivation Tangible/material support Emotional support Strong relationship to program staff Social component
5. What factors might prevent your child and you from participation in a weight management program?	Time-related Convenience-related (scheduling/conflicts) Transportation Lack of progress Lack of support Cost-related Motivation-related Negative emotions	5. What sorts of things could your doctor or a health coach teach you to help you have a healthy weight?	General knowledge about health/diet/exercise Skills/tools Education around habit/behavior change and strategies Combining education with actual strategies for change
		6. What would prevent you from being in a healthy weight program with your doctor or with a health coach?	Scheduling conflicts/busy – getting sick/emergencies Lack of confidence/support Not feeling prepared/supported (external) Low motivation (internal) Stigma, getting bullied Program is boring Cost
6. If we are successful in developing curriculum and materials for a family-based weight management program, what would that look like?	Digital/application-based Individualized Support Incentives/motivation Tips/resources, education Behavioral prescriptions Culturally appropriate	7. What sorts of fun activities would you want to do with your doctor or health coach?	Competitive games Team games Novelty/general fun
		8. What would help make sure you and your parents came to see your doctor or health coach every couple of weeks?	Fun (novel) Results (progress visible) Incentives (rewards) Good relationship with coaches (welcoming/enjoyable)

Although all groups reported weight loss and weight maintenance as among the most important results they would expect from a WMP for children/adolescents, parents also stated they would expect cognitive, affective, and behavioral changes, such as increased self-esteem, increased knowledge about health behaviors, behavior change (eg, “healthy eating” and “increased activity”), and improved long-term health and lifestyle change.

Parents across groups reported that they would want to stick with a program if they were seeing progress or positive results (eg, “losing weight” and “feeling good”). Parents also reported that they would be more likely to stick to a program if it was accessible, fun, and interesting to their children/adolescents. Parents also reported that they would continue participating if the program provided tangible incentives, social support, and motivating coaches.

When asked to identify factors that might prevent them from participating in a program, parents reported factors related to time, scheduling conflicts, convenience, transportation, cost, motivation, and affect/emotion. Motivation-related factors included fear, embarrassment, or judgment and low motivation stemming from prior failures when attempting to lose weight. Parents also mentioned that the presentation of the program “is key,” including the name of the program and who the program is presented by, with some parents emphasizing that they would likely not participate if the program was not accommodating to health or disability issues and if it was not culturally sensitive. Parents across groups also reported that perceiving a lack of support and not seeing progress or results might also prevent them from participating.

Regarding important factors they would want included in a WMP for children/adolescents, parents thought a WMP should include educational components, involve skill-building and behavior change strategies, have incentives, be fun and positive, family-oriented, and culturally appropriate. The educational components suggested by the parents varied from general health education about the effects of diet, sleep, and excess weight on overall health to the nutritional components of foods and recommended portion sizes. Parents also noted that they would want a WMP to include social support (eg, “peer to peer support” and “community support”) and coaching (eg, “life coaches” and “knowledgeable instructors”); that the program would need to be accessible, feasible, sustainable, and affordable; and that mobile or technological components could facilitate the program. These same themes were echoed in the responses to the question of what parents expected a successful WMP to look like.

#### Children/Adolescent Responses

Children/adolescents reported several ways that their parents, doctors, or teachers currently help them manage their weight, including providing education (eg, a teacher “talks to us about eating healthy and eating vegetables”); feedback (eg, “they can tell you what you’re doing wrong”); encouragement and advice regarding healthy practices; monitoring their weight and helping them engage in activity and eat healthy foods; and limiting consumption of unhealthy foods.

Children/adolescents across the focus groups identified 3 major types of challenges that they face with maintaining a healthy weight: bullying, physical limitations, and psychological/behavioral struggles (eg, lack of knowledge, motivation, self-confidence, and self-control [“You think you can’t change the way you are”] and lack of environmental control, such as family get-togethers, where unhealthy food is served).

Visible results were among the most-mentioned themes that children/adolescents said they would want to see in a program, but they also noted that they would want improved physical and psychological health, including improved fitness and increased self-esteem. Reduced bullying was another important result that children/adolescents across groups wanted. Children/adolescents also wanted to achieve successful adoption of healthy behaviors, including increasing their knowledge of healthy living, trying new healthy foods, and incorporating healthy habits into daily routines.

Children/adolescents reported that they would want to stick to the program if they saw physical results, learned new skills, gained knowledge about healthy living, and achieved better health overall. They also mentioned that a program that is fun and doesn’t feel like school would make them more likely to stick with it. Children/adolescents stated that they would stick with a program if it had social components and if they perceived a strong relationship to program staff. These themes were echoed in the children/adolescent responses to the question of what factors would help them continue to attend the program sessions regularly. In particular, they said they would continue to be engaged if the program included incentives or rewards for healthy behaviors and fun and novel activities, including competitive or team games, if they had a good relationship with the health coach, and if they achieved visible progress.

Obstacles that would prevent children/adolescents from participating in the program were related to internal and external barriers. External barriers to participation were scheduling conflicts, cost of the program, lack of support or feeling unprepared (eg, “if you have to do it alone”), bullying, and a boring program that felt like a doctor's appointment. Internal obstacles that might prevent continued engagement in a WMP were lacking confidence or motivation and feeling embarrassed or stigmatized from bullying.

Both parents and their children/adolescents perceived that the program should be motivating, appealing, fun and educational, diverse, culturally appropriate, sustainable, and involve the entire family.

## DISCUSSION

In this study, we examined the perceptions of parents, the majority of whom were African American, and their children/adolescents concerning the components that should be included in a family-based WMP adapted for a PCP setting and the perceived preferences, benefits, and barriers to participation. Although parents and their children/adolescents participated in separate focus groups, similar responses were expressed, apart from a few expected age-related differences. For example, during the preliminary questioning, all participants perceived that an expected result in a WMP is visible or tangible weight loss, which aligns with other research suggesting that participants may be inclined to expect that a WMP has a focus on physical results.^37^ However, parents and children/adolescents also both identified improved psychological well-being as a desired program outcome, including increased self-esteem, reduced bullying experiences, and improved self-confidence. These responses suggest that psychological well-being and physical health should be important targets of a family-based WMP.

Additionally, activities with family and friends that are fun and a team that encourages and provides support were viewed as necessities for participation in a family-based WMP. Research has shown that when an activity is fun and performed in a supportive atmosphere, participants are likely to remain in the study.^37^ Fun has also been identified in qualitative research as an enabler of program participation, particularly by adolescents.^25^ All groups overwhelmingly conveyed a desire for healthy vs unhealthy eating, a list of healthy foods including recipes, and cooking demonstrations to show that healthy eating is important and approachable. These similarities concur with research in which parents and children/adolescents with overweight and obesity expected that they would lose weight, especially if they were physically active and eating in a more healthy way^38^ and with research demonstrating that participants are most successful when they are shown how to implement healthy behaviors in addition to being told which behaviors to change.^39,40^

A major obstacle perceived by participants that would prevent participation in a WMP was time (parents’ work schedules and kids’ extracurricular activities), a finding that echoes previous research.^26^ In contrast, parents expressed concern about access to available resources and cost of the program, while children/adolescents expressed concerns about bullying preventing participation in a WMP. Multiple studies suggest that bullying is significantly associated with low self-esteem in overweight children/adolescents.^41,42^ Additionally, research has shown that differential participation might not result from a person's lack of willingness to participate but may be attributable to other causes such as time, access, or financial resources; these barriers may influence how researchers approach recruitment of populations that have been traditionally underrepresented in medical research.^43-47^

Parents commonly reported that if researchers were successful in developing curriculum and materials for a family-based WMP, these resources should be available online and/or accessible on a smartphone, suggesting that program delivery should be flexible. Thus, parents and their children/adolescents would have access to the program and the ability to participate when, where, and how at their convenience. Emerging evidence suggests that the best outcomes derive from multidisciplinary delivery approaches that use a broad range of expertise and varied interventions with proven collaboration.^48^

Parents across all groups perceived that the 3 most important strategies having the greatest impact in a family-based WMP would be education on healthy vs unhealthy eating, parental involvement, and effective program leaders, with parental involvement identified as the easiest to implement. Parental and/or familial involvement was critical for both parents and children/adolescents, as they expressed the need for support from each other for success and sustainability, as well as support from effective program leaders. To maintain long-term behavior change, children/adolescents with overweight and obesity and their parents need support from their extended family, school, friends, peers, and their PCP.^38^

Likewise, children/adolescents perceived that education on healthy vs unhealthy eating and exercise would have the greatest impact in a WMP, with healthy eating and meal plans identified as easiest to implement. Research has shown that nutritional and dietary consultation is one of the most useful supplementary services available in clinical practice for weight management.^49,50^

This study has several strengths. An advantage to using the NGT is that the weight of each participant's opinion is the same, and process loss seems less likely to occur.^51^ The highly structured format of the NGT provides an opportunity for group participants to achieve a substantial amount of work in a relatively short period of time. The NGT deliberately avoids interpretation from a facilitator who has the responsibility to explore but not interfere with or influence participants in the group.^27^

The NGT also has limitations: the composition and representativeness of participants may limit the generalizability of the results, training and preparation are required, the discussion is restricted to a single question, and further elaboration of other ideas is not allowed.^52^ However, coding and identification of common themes across the focus groups allowed for interpretation of other responses to complement the NGT findings. This study is further limited by its convenience sampling of available parents and their children/adolescents.

Overall, the results of this study suggest that parents and children/adolescents have similar perceptions and expectations of family-based WMPs in the primary care setting as they do of WMPs in other settings and face similar barriers. This study's findings also highlight the importance of psychosocial factors in family-based WMPs. Both parents and children/adolescents identified physical health and psychological well-being as desirable program results, suggesting that increasing self-esteem, helping adolescents cope with bullying, and helping improve overall mental well-being could be important targets in a WMP.

Future research exploring the design and implementation of FBTs for pediatric obesity in primary care settings could benefit from this study's findings. However, the present study also identifies several challenges. Delivering a high-quality WMP that is also fun, accessible, and supportive may be difficult for PCPs who are overburdened and in short supply.^53^ Similarly, future research will need to demonstrate the feasibility and cost-effectiveness of such programs.

## CONCLUSION

Knowledge (education on healthy vs unhealthy eating), parental involvement that engages family and friends in activities such as dancing and games that are fun, and a knowledgeable team (effective program leaders) that provides incentives and support are the ingredients that parents and children/adolescents reported as necessary for success in family-based WMPs implemented in primary care. These findings may assist researchers in designing and planning sustainable interventions in future studies.

## Notes

This article meets the Accreditation Council for Graduate Medical Education and the American Board of Medical Specialties Maintenance of Certification competencies for Patient Care, Medical Knowledge, and Practice-Based Learning and Improvement.
